# Clinical and structural disconnectome evaluation in a case of optic aphasia

**DOI:** 10.1007/s00429-024-02818-z

**Published:** 2024-06-25

**Authors:** Laura Veronelli, Rolando Bonandrini, Alessandra Caporali, Daniele Licciardo, Massimo Corbo, Claudio Luzzatti

**Affiliations:** 1 Department of Psychology, University of Milano-Bicocca and Milan Center for Neuroscience, Milan, Italy; 2 Department of Neurorehabilitation Sciences, Casa di Cura IGEA, Milan, Italy; 3 Department of Medicine and Surgery, University of Milano-Bicocca, Monza, Italy

**Keywords:** Optic aphasia, Associative visual agnosia, Occipital lobe, Semantics, Splenial disconnection, Disconnectome

## Abstract

Optic Aphasia (OA) and Associative Visual Agnosia (AVA) are neuropsychological disorders characterized by impaired naming on visual presentation. From a cognitive point of view, while stimulus identification is largely unimpaired in OA (where access to semantic knowledge is still possible), in AVA it is not. OA has been linked with right hemianopia and disconnection of the occipital right-hemisphere (RH) visual processing from the left hemisphere (LH) language areas.

In this paper, we describe the case of AA, an 81-year-old housewife suffering from a deficit in naming visually presented stimuli after left occipital lesion and damage to the interhemispheric splenial pathway. AA has been tested through a set of tasks assessing different levels of visual object processing. We discuss behavioral performance as well as the pattern of lesion and disconnection in relation to a neurocognitive model adapted from Luzzatti and colleagues (1998). Despite the complexity of the neuropsychological picture, behavioral data suggest that semantic access from visual input is possible, while a lesion-based structural disconnectome investigation demonstrated the splenial involvement.

Altogether, neuropsychological and neuroanatomical findings support the assumption of visuo-verbal callosal disconnection compatible with a diagnosis of OA.

**Supplementary Information:**

The online version contains supplementary material available at 10.1007/s00429-024-02818-z.

## Introduction

The term optic aphasia (OA) was introduced by Carl Samuel Freund in 1889 to describe patients suffering from right hemianopia and impaired naming of objects on visual presentation, although they could identify the same objects on sight, name them after tactile manipulation and were sometimes able to demonstrate the use of the unnamed object via pantomime. One year later, Lissauer (1890) reported the occurrence of a modality-specific visual disorder that he called associative visual agnosia (AVA), caused by a primary damage to the visual identification of objects, but spared identification and naming of the same objects after tactile manipulation. In this context, the core distinction between identification and naming concerns the fact that identification corresponds to “knowing what the object is”, while naming corresponds to “knowing how the object is named”, after retrieving the object semantic information.

Freund explained OA in terms of visuo-verbal disconnection, due to both a left parieto-occipital lesion determining right side hemianopia, and a splenial lesion causing a disconnection of the intact right occipital lobe from the left hemisphere (LH) language areas. However, this explanation has not been unanimously accepted (Freud 1891; Goldstein 1906; Kleist 1934; Wolff 1904): for instance, a similar pattern of anatomical and functional damage has been proposed also for AVA (e.g. Geschwind 1965). As for OA, in AVA (Lissauer, 1890), unilateral lesions involve the left occipital cortex, the left inferior longitudinal fasciculus, as well as the callosal splenium (but see the case of AVA described by McCarthy and Warrington 1986; in which the splenium was apparently spared; see also Ptak et al. 2014; Feinberg et al. 1994; and Gainotti and Marra 2011; for reviews on AVA anatomical underpinnings).

Different functional interpretations of OA were proposed in the cognitive neuropsychological literature: as a disconnection between visual and semantic systems (Beauvois 1982; Lhermitte and Beauvois 1973), as “access visual agnosia” (Hillis and Caramazza 1995; Leek et al. 1994; under the label “visual modality-specific naming impairment”; Riddoch and Humphreys 1987), as a disruption of a non-semantic route for naming (Ratcliff and Newcombe 1982), or as an additive access deficit of the semantic system and the phonological output lexicon (Campbell and Manning 1996; Farah 1990; Manning and Campbell 1992; Raymer et al. 1997; Sitton et al. 2000).

The concept of a visuo-verbal disconnection was then successively reintroduced by Coslett and Saffran (1989, 1992): following a left occipital lesion, visual information has to be processed in the right hemisphere (RH) and activate semantic properties in the isolated RH. This assumption was based on the preserved performance of patient EM in categorization and associative matching tasks. However, RH information could not be transferred to LH language areas because of the callosal interruption. Thus, the visual naming deficit observed in such patient was attributed to the inability to access the LH phonological output lexicon from RH semantics.

In 1998, Luzzatti and colleagues further specified the concept of right and left hemisphere semantics while describing patient AB, who displayed the typical OA performance pattern, with impaired visual object naming, as well as spared tactile naming and naming to definition. In this case the lesion involved the left occipital inferior and mesial cortex and the underlying white matter, in the vascular territory of the left posterior cerebral artery. Luzzatti and colleagues (1998) interpreted AB’s difficulties as caused by a disconnection of RH visual semantics from LH verbal semantics. In this view, visual semantics would be represented bilaterally and symmetrically in the brain, whereas verbal semantics would be predominantly organized in the LH only. Accordingly, the RH would contain some phonological and orthographic input lexical information, although almost only for high-frequency concrete words (e.g., Bonandrini et al. 2020; Schweiger et al. 1989; Semenza and Luzzatti 2019; Zaidel 1991). This interpretation has been systematized in a model of oral and written language processing (Luzzatti et al. 1998, 2003), that portrays the cognitive functional operations carried out by the two cerebral hemispheres.

As far as the differential diagnosis between OA and AVA is concerned, the core difference between OA and AVA lies in the fact that in AVA semantic access from visual input is completely impaired, while in OA semantic access from visual input is still possible: the impairment rather lies in the retrieval of the target lexical labels (Gerlach and Robotham 2021; Luzzatti et al. 1998).

In a complementary neuroanatomical fashion, Schnider and colleagues (1994) proposed a diagnostic criterion based on the extent of callosal damage: the involvement of the splenium - and more specifically, the disconnection of left inferior temporo-occipital areas from the RH visual input - would only underlie OA. If these connections are spared to some degree, the LH would maintain its processing advantage over the RH. The pattern would then be consistent with AVA, with the severity of the deficit depending on the extent of the left inferior temporo-occipital lesion^1^. This apparently counterintuitive relationship between the severity of the deficit (AVA > OA) and the entity of the lesion relies on the hypothesis that, according to Schnider and colleagues (1994), only a full splenial disconnection allows RH compensation for the LH lesion.

Still, the distinction between AVA and OA proves difficult, vague or impossible to evince in some cases, and some patients reported in literature showed intermediate patterns or shift from one clinical condition to the other (Barca et al. 2009; Chanoine et al. 1998; Schnider et al. 1994). Also from the anatomo-pathological point of view, a similar LH lesion pattern involving the occipital lobe seems to cause either right homonymous hemianopia (RHH), or RHH and pure alexia, or RHH, pure alexia, and color anomia, or RHH, pure alexia, color anomia, and OA, or RHH, pure alexia, and AVA (Binder and Mohr 1992; Damasio and Damasio 1983; De Renzi 1999; De Renzi et al. 1987; De Renzi and Saetti 1997; Mohr and Binder 2011; Schnider et al. 1994). These limitations in differential diagnosis are further complicated by the lack of a comprehensive neurocognitive model adopted as reference point to interpret behavioral performance with reference to the neural damage.

In this study, we report the clinical pattern observed in case AA, a patient with a neuropsychological deficit consistent with that of OA. A set of tasks assessing different visual object processing levels were administered and a lesion-based structural disconnectome study was performed, in order to define the involvement of specific inter- and intra-hemispheric tracts. The case is reported from the angle of clinical differential diagnosis between OA and AVA (Gerlach and Robotham 2021).

**Fig. 1:**
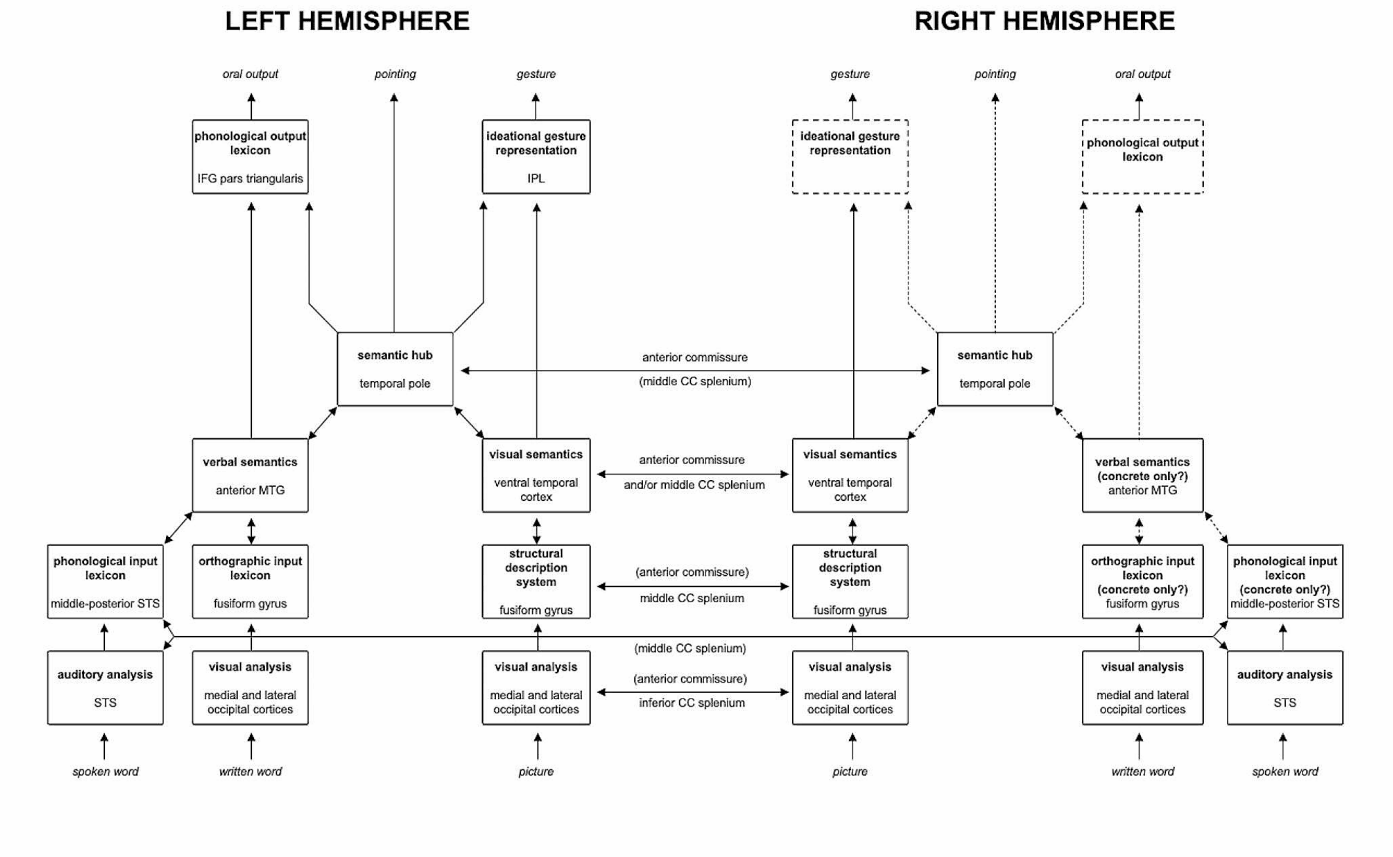
Neurocognitive model of the pathways for object identification and naming in the left and right hemispheres. Dashed lines indicate modules and connections for which there is little (if any) evidence in the RH. Please note that the link between the semantic hub and pointing is underspecified and most likely mediated by other brain regions. STS = superior temporal sulcus; MTG = middle temporal gyrus; IFG = inferior frontal gyrus; IPL = inferior parietal lobule

**Fig. 2:**
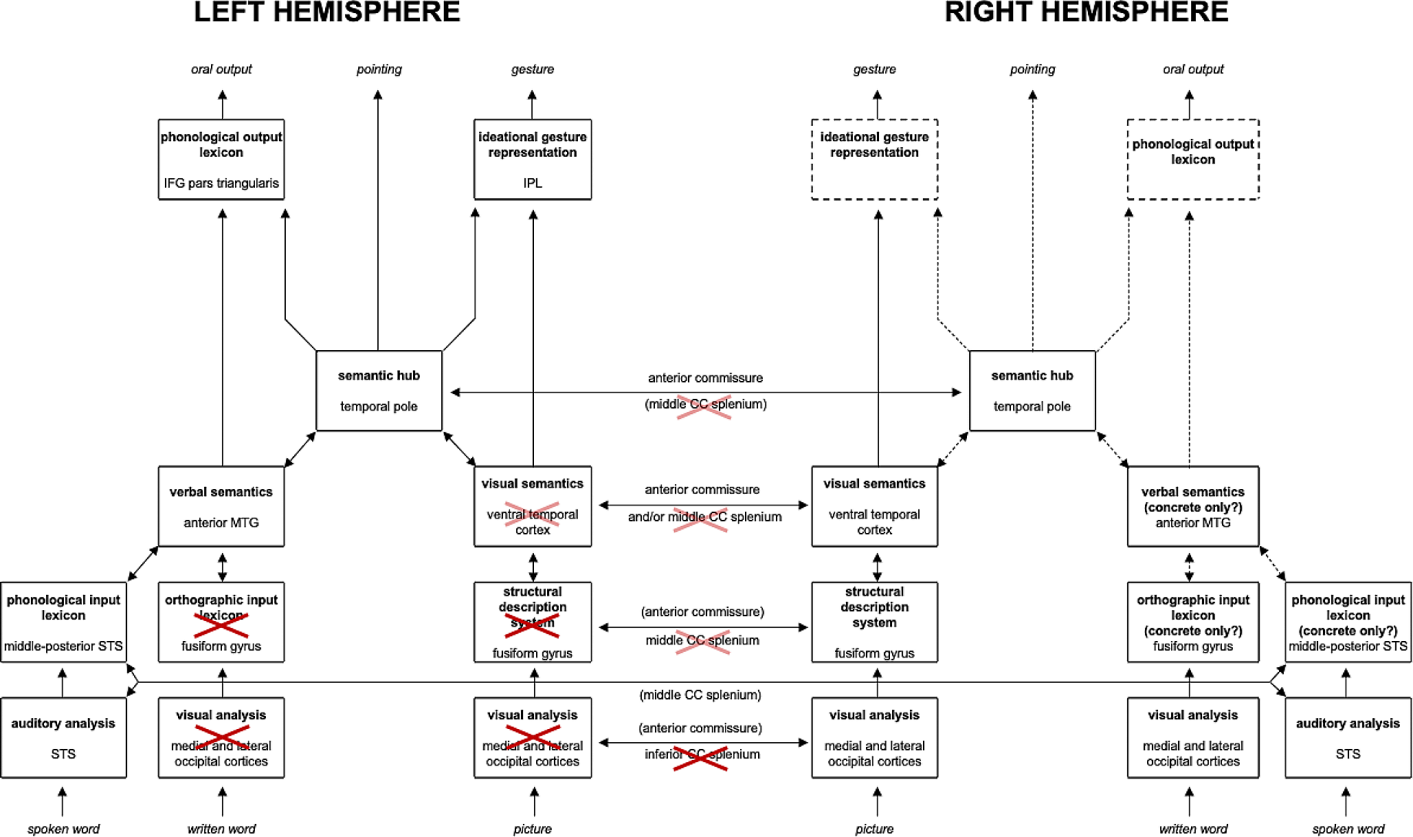
Graphical representation of the patient’s lesion and disconnection profile, with reference to the neurocognitive model of the pathways for object identification and naming in the left and right hemispheres. Opaque Xs represent lesions and disconnections; semi-transparent Xs indicate neural units and structural connections that may not be completely impaired in patient AA. In AVA, access to semantic representations from visual input, possible in OA, would be impaired (Gerlach and Robotham 2021), due to lesion of / disconnection from its neural substrates. This would constitute the critical difference between the two conditions (color online and in PDF)

The main aim of the study is to discuss and integrate cognitive performance and structural disconnectome findings, in the light of an updated neurocognitive version of the model proposed by Luzzatti and colleagues (Luzzatti et al. 1998; Luzzatti 2003) (see Figs. 1 and 2) for OA. Identical to the original description of the model are the general outline of the modules, the subdivision into left and right hemispheres, and the assumption that verbal abilities in the RH are only limited to concrete and frequent items. In this version of the model the overall direction of the flow is bottom-up to mimic the direction of processing from occipital (input) to frontal (output) cortices, and a new module has been added to accommodate recent evidence suggesting the existence of a cross-modal “semantic-hub” (Patterson et al., 2007). A tentative set of neural correlates of the different components of the model (according to available literature) have also been added (see Supplementary Table S4, for references and Figure S1 for an overview of structural connections).

## Case report

**Fig. 3:**
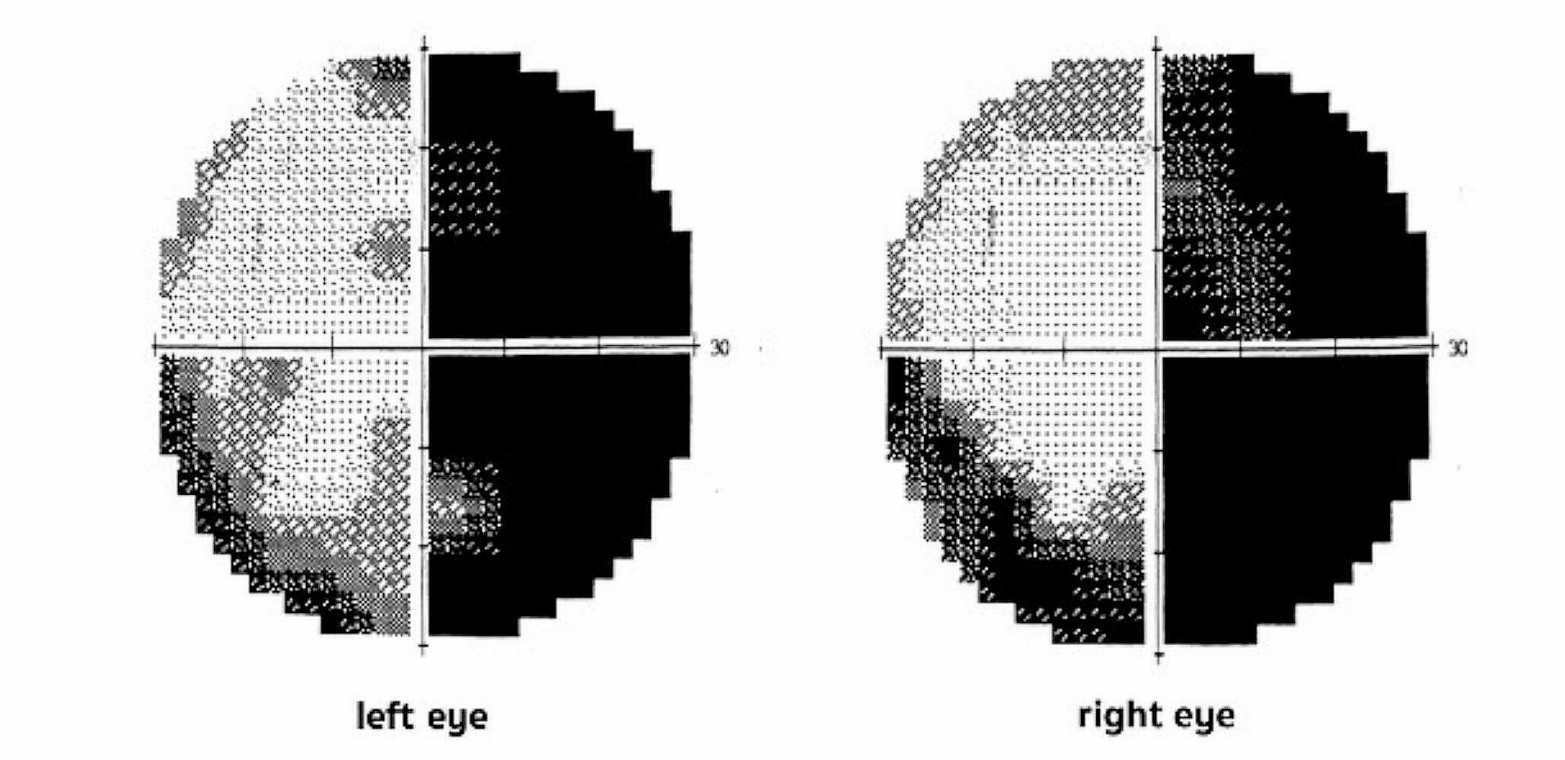
AA’s visual field perimetry, demonstrating the presence of right hemianopia after left occipital lesion

AA is an 81-year-old right-handed housewife with 8 years of education, who was hospitalized for a stroke involving the left occipital and temporo-occipital inferior and mesial cortical and subcortical structures, due to occlusion of the left posterior cerebral artery. Ten days after the event, she was hospitalized to take part in a neurorehabilitation program: at a first neurological examination, she presented with mild right hemiparesis; Humphrey’s visual field perimetry revealed right homonymous hemianopia, which was acknowledged but not completely compensated by the patient (Fig. 3). A clinical neuropsychological assessment revealed the presence of alexia without agraphia (see Bonandrini et al. 2020; for a detailed description of the reading impairment) and naming deficits limited to the visual modality. The Aachen Aphasia Test (AAT, Huber et al. 1984; Luzzatti et al., 2023, 2024) revealed that spontaneous speech was only mildly impaired, showing very slight difficulties in lexical access (score 4/5), with no additional phonological, morphosyntactic or articulatory and prosodic impairment. Reading aloud was severely impaired (1/30; Percentile Rank, PR: 13), as well as naming from visual presentation (moderate impairment: 55/120; PR: 38), producing, among other errors, several perseverations and verbal paraphasias (see Table 1).

The following section addresses the investigation of AA’s identification and naming abilities, performed during her hospitalization, aimed at detecting the impaired functional components underlying AA’s naming impairment. The study was approved by the local Research Ethics Committee (Comitato Etico Milano, Area 2; ID 819), and informed written consent was obtained from AA, according to guidelines established by the 1964 Declaration of Helsinki.

**Table 1:** Scores obtained by AA at the Aachen Aphasia Test (AAT, Huber et al. 1984; Luzzatti et al. 2023, 2024) at the first neuropsychological assessment (two weeks after stroke)

AAT subtest	Item set/Observation level	Range	Score	PR	Severity
*Spontaneous speech*	Communicative behavior (COM)	0–5	4		
	Articulation and prosody (ART)	0–5	5		
	Formulaic automated language (AUT)	0–5	5		
	Lexical and Semantic structure (SEM)	0–5	4		
	Phonemic structure (PHO)	0–5	5		
	Syntactic structure (SYN)	0–5	5		
*Token Test (TT)*		50 − 0*	22	65	Mild-to-moderate
*Repetition (REP)*		0 -150	149	100	Minimal-to-no impairment
*Written language (WRIT)*	Reading aloud	0–30	1	13	Severe
	Composing words/sentences from graphemes/morphemes	0–30	11	45	Moderate
	Writing to dictation	0–30	21	75	Mild
*Naming (NAM)*		0–120	55	38	Moderate
*Comprehension (COMP)*	Auditory comprehension	0–60	48	64	Mild-to-moderate
	Reading comprehension	0–60	0	2	Severe

PR: Percentile Rank; * In the subtest Token Test (TT), the number of errors is counted

## Neuropsychological testing

AA’s visual, lexical and semantic abilities were investigated following the procedure described by Luzzatti and colleagues (1998).

*Section A: visual and tactile naming*. AA was asked to name 25 visually presented real manipulable objects (artifacts) that she saw in a prototypical perspective, and she was not allowed to touch. In a following session, she had to name the same objects after left-hand haptic exploration out of vision. A time limit of 10s was given for each item. AA’s performance on the object naming task for tactile exploration was significantly better than that on visual presentation (22/25 and 12/25, respectively; χ^2^(l) = 9.19; *p* < .01) (see Table 2A). A qualitative analysis of the naming responses after visual presentation revealed that the patient produced 10 perseverations, 1 semantic paraphasia, 1 semantic paraphasia/visual error and 1 visual error. In particular, perseverations were related, but not necessarily identical to previous responses. For example, she correctly named item 5 “pencil sharpener”, but then she answered: (i) item 7, tweezers → “pencil sharpener”; (ii) item 9 hammer → “used to sharpen the pencils”; (iii) item 14 bottle opener → “blade for sharpening pencils”; (iv) item 15 battery (electric pile) → “pencil sharpener”; (v) item 19 phone token (used in the past on public phones) → “pencil sharpener of the past” (this “of the past” specification suggests access to semantic features that are specific of the target item). After tactile presentation, she produced 2 verbal paraphasias and 1 efficacious circumlocution.

When tested with line drawings, AA’s oral naming abilities resulted to be impaired on both object and verb action naming (Batteria per l’Analisi dei Deficit Afasici, BADA, Miceli et al. 1994), with no difference between nouns and action verbs (23/30 and 20/28, respectively; χ^2^(l) = 0.21; *p* = .65). She also performed poorly on a living and non-living object naming task (15/48, Catricalà et al. 2013), without difference between living (9/24) and non-living (6/24) items (χ^2^(l) = 0.87; *p* = .35). On the other hand, AA’s performance on naming to definition (Novelli et al.,1986) fell within the normal range (36/38).

In this section, AA showed a naming deficit that is specific to the visual modality: she performed better on naming after haptic exploration and to definition, which is compatible with either OA or AVA.

In the next sections, each step of visual object naming, from early visual analysis to oral output, has been addressed.

**Table 2:** Performances exhibited by AA in a set of tasks assessing each step of visual object processing (one month after stroke)

**Section A: visual and tactile naming**	Raw score/number of items	Adjusted score	Cut-off	Equivalent score° / Percentile Rank
*Naming real objects: visual presentation (ad-hoc task)*	12/25#			
*Naming real objects: haptic presentation (left hand; ad-hoc task)*	22/25			
*Naming of line drawings*				
Oral object naming - BADA (Miceli et al. 1994)	23/30*		28	
Oral action verb naming - BADA (Miceli et al. 1994)	20/28*		27	
Living and non-living object naming (Catricalà et al. 2013)	15/48	15.6		0*
*Naming to definition (*Novelli et al. 1986)	36/38	36		2
**Section B: visual analysis**				
*Birmingham Object Recognition Battery (BORB;* Humphreys and Riddoch 1993)				
Length match task	25/30		24	
Size match task	25/30		23	
Orientation match task	26/30		20	
*Poppelreuter-Ghent’s test - objects (*Della Sala et al. 1995)	18.22	17.92		0*
*Poppelreuter-Ghent’s test - abstract line drawings (*Della Sala et al. 1995)	7.75	8.05		0*
*Copy of line drawings (*Spinnler and Tognoni 1987)	5/14	5.6		0*
**Section C: access to the structural description system**				
*Object decision task (line drawings of real and chimeric animals;* Luzzatti et al. 2020)	30/34		28	
**Section D: from an object name to the underlying visual representations**				
*Oral comprehension of nouns - AAT (*Luzzatti et al. 2024)	26/30			PR = 66
*Oral comprehension of sentences - AAT (*Luzzatti et al. 2024)	22/30			PR = 52
*Oral comprehension of nouns - BADA (*Miceli et al. 1994)	38/40*		39 (mild deficit)	
**Section E: access to visual semantics**				
*Picture-to-picture matching*				
Pyramids and Palm Trees Test (Gamboz et al. 2009)	35/52	36.25*	40.15	
Semantic Association Test (Luzzatti et al. 2020; Banco et al. 2023)	41/76	42.09		0*
*Sorting pictures into categories (ad-hoc task)*	32/32			
**Section F: color naming and object color knowledge**				
*Color naming - AAT (*Luzzatti et al. 2024)	17/30*		(moderate deficit)	37
*Name-to-color matching*	5/5			
*Object-Color Knowledge (ad-hoc task)*	40/40			
**Section G: limb and oral apraxia**				
*Ideomotor apraxia (De Renzi et al.,* 1980) *(right hand)*	41/72*		52	
*Ideomotor apraxia (De Renzi et al.,* 1980, 1996) *(left hand)*	43/72*		52	
*Pantomime after visual presentation of objects (ad-hoc task)*	7/15			
*Pantomime after verbal presentation of objects (ad-hoc task)*	15/15			
*Oral apraxia (*Spinnler and Tognoni 1987)	15/20	15.25		0*

# Errors were predominantly perseverations

° Following Capitani and Laiacona’s (1997) and Spinnler and Tognoni’s (1987) procedure, adjusted scores of neurologically healthy participants were classified into five categories (“equivalent scores”, from 0 to 4). Zero corresponds to a score below the 95% tolerance limit at 95% confidence level. Four corresponds to a score that is ≥ of the median value, and 1, 2, 3 are intermediate values between 0 and 4

* Pathological performance according to available normative data

*Section B: visual analysis.* AA’s ability to produce an on-line representation of visual stimuli was assessed by: (i) the Length, Size and Orientation match tasks of the Birmingham Object Recognition Battery (BORB, Humphreys and Riddoch 1993); (ii) the Poppelreuter-Gent overlapping figures test, where she had to identify three to five overlapping line drawings by pointing to each of the target drawings among 10 individual alternatives displayed underneath the overlapped images (Della Sala et al. 1995); (iii) the copy of geometric line drawings (Spinnler and Tognoni 1987). When tested on the BORB Length, Size and Orientation match tasks, the patient performed within the normal range. Her performance on the Poppelreuter-Ghent’s test was impaired for objects and abstract line drawings (predominant choice of left-side elements); her ability to copy geometrical line drawings was also mildly impaired (Table 2B). In these latter tasks, a qualitative error analysis showed one omission and one deformation on the right side of the stimuli, together with a tendency to copy figures more to left with respect to the objective midpoint of the sheet. We suggest that these data could be attributed to the presence of right hemianopia, which was not completely compensated by the patient, or to the additional presence of a mild right spatial neglect. Indeed, when tested through visuo-spatial tasks, she performed flawlessly at the line cancellation test (Albert 1973), but she exhibited 1 left-sided and 7 right-sided omissions at the Bell cancellation test (Gauthier et al. 1989), performing outside the established cut-off score (Vallar et al. 1994). Furthermore, she showed a large deviation to the left with respect to the objective midpoint of the stimulus in the Line bisection task and left position preference on the Raven’s Progressive Matrices test (Colombo et al. 1976).

In general, although AA’s performance was influenced by the presence of hemianopia and possibly right spatial neglect, her early visual processing abilities were substantially preserved.

*Section C: access to the structural description system.* After early visual processing, the episodic representation obtained from the image of an object needs to match its corresponding stored structural representation to allow identification (see Humphreys and Riddoch 1993; Marr 1980). To tap this function, AA was asked to decide whether items depicted by a line drawing (real animals or chimeric images, namely non-real animals consisting of two different types of animals, e.g., half camel/half giraffe, half cat/half chicken) were real animals or not. In this task she performed within normal range (Table 2C).

*Section D: from an object name to the underlying visual representations.* Word-to-picture matching tasks were used to investigate the relationship between words and pictures. AA showed a mild-to-minimal deficit in both the oral word comprehension task of the AAT (26/30; Luzzatti et al., 2023, 2024); and the oral noun comprehension task of the BADA (38/40; Miceli et al. 1994; Table 2D).

**Fig. 4:**
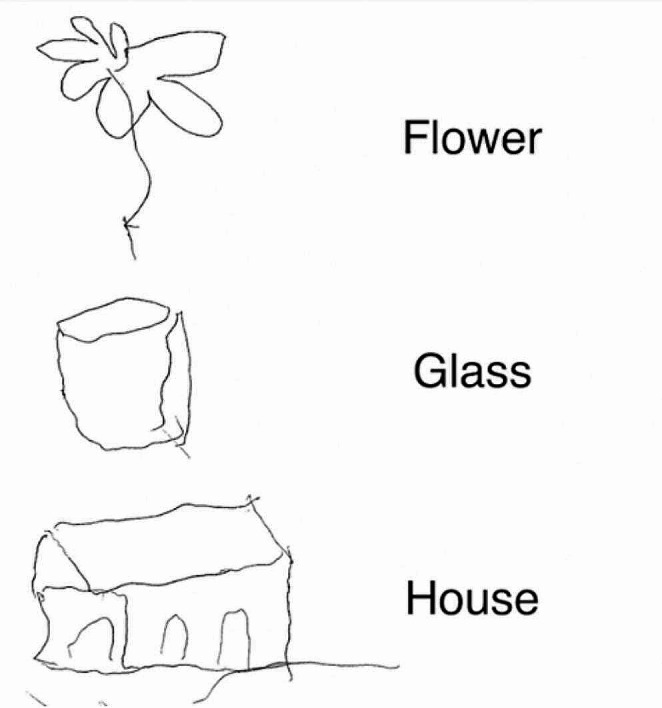
Example of AA’s drawings from memory

Figure 4 shows AA’s performance in drawing from memory, demonstrating, beyond some slight deformations on the right side, the global preservation of AA’s access to stored visual representations. Ten healthy subjects (7 F, 3 M; mean age = 32.3 years, SD = 4.32, range 26–39, and mean education = 18.70 years, SD = 2.31, range 15–22) correctly recognized AA’s drawings from memory (100% agreement, except for the drawing of a “glass”, whose agreement was 90%).

*Section E: access to visual semantics.* This section aimed at assessing the integrity of the access to semantic knowledge from structural description. In order to test AA’s access to semantics from pictures we used the Pyramids and Palm Trees Test (PPT) developed by Howard and Patterson (1992). Fifty-two picture triads were shown to AA, one at the top and two at the bottom of an A4 sheet. She was asked to select, through finger pointing, which of the two bottom items was semantically most related to that on the top. The score obtained by the patient (35/52) was below the lower tolerance limit (Gamboz et al. 2009; for normative data in Italian). Visual semantic memory was further assessed through the Semantic Association Test (SAT; Banco et al. 2023; Luzzatti et al. 2020), using a picture-to-picture matching paradigm. Once again, AA obtained a pathological score (41/76). However, performance in these tests could be invalidated by AA’s scarcely compensated right hemianopia and additional spatial neglect, since a qualitative analysis of errors indicated that, out of 35 errors on the SAT test, the patient committed 25 mistakes out of 38, choosing the picture on the left instead of the right side, and 10 errors out of 38, choosing the picture on the right instead of the left side (Fisher’s exact test, two-tailed: *p* = .001). Furthermore, it is worth mentioning that in a picture-to-picture matching task a participant may not necessarily employ a purely visual strategy bypassing language, as usually thought. Despite the purely visual characteristics of the stimuli, the task may become easier when a lexical-semantic association is used rather than a visual association of the two objects in a same context. For instance, when neurologically healthy individuals see a pyramid, they may associate this image with the lexical-semantic concept of Egypt and the concept of Egypt with a tuft of palm trees (“Pyramids are in Egypt – also palm trees are typical of Egypt”). Therefore, AA’s poor performance on the picture-to-picture matching task may be also interpreted as the result of a tentative impaired access to lexical and/or lexical-semantic knowledge from vision (Luzzatti et al. 1998; Luzzatti 2003).

Furthermore, spared semantic access was demonstrated by AA’s preserved ability in sorting pictures into categories, when the names of categories were supplied by the examiner, similarly to what occurred for patient AB (Luzzatti et al. 1998). When presented with 32 pictures, one at a time, and asked to categorize each item according to three classes (animals, vegetables, and tools), she performed flawlessly (score 32/32; see Table 2E).

In this section, AA showed evidence of spared semantic access from visual input that should be typically impaired in AVA (Bartolomeo 2022).

*Section F: color naming and object color knowledge.* Color naming is classically impaired in OA. AA was asked to name 10 patches of prototypical colors (the color naming section of the AAT), scoring 17/30. Errors were 3 perseverations and 2 semantic paraphasias (other color names). When she was asked to point to a color patch named by the examiner from 5 alternatives, she performed flawlessly (5/5). Object color knowledge was assessed by asking the patient to retrieve from memory the typical color of black and white line drawings. AA was given 40 items, 22 natural (fruits, vegetables) and 18 artificial objects (conventional colors of artifact objects, e.g., a fire truck = red). When AA was asked to give a verbal response (the name of the corresponding color), she performed flawlessly (Table 2F). The discrepancy found between color naming and object color knowledge is in line with pure lexical damage from visual stimuli that also extends to color names (see Siuda-Krzywicka et al. 2019; for a similar dissociation between color naming and color categorization).

*Section G: limb and oral apraxia.* AA’s abilities on visual imitation of meaningful and meaningless gestures were assessed through the limb-apraxia test devised by De Renzi et al. (1980) and an oral apraxia test (Spinnler and Tognoni 1987) (Table 2G). She presented deficits in motor programming for both the upper limb and oral districts. Her pantomime after visual presentation of an object, carried out with her right hand, was impaired (7/15), but she performed flawlessly on pantomime after verbal command (“show me how to use a hammer”).

*Interim discussion*. We described the case of a patient, AA, suffering from modality-specific deficit in naming line drawings and real objects from sight with much better tactile naming and spared naming to definition (Section A). Her naming deficit from visual modality was not caused by early identification problems, since AA was able to analyze visually presented stimuli adequately (Section B). Seemingly, unimpaired performance on the reality decision task indicates that AA was able to access the structural description of objects (Section C). AA performed flawlessly on a word-to-picture matching task and was able to draw objects from memory from verbal stimuli (Section D). The relatively accurate performance on these tasks indicates that AA was able to process visual knowledge of objects from verbal stimuli. Her minimal impairment on a word-to-picture matching task is consistent with that reported by Luzzatti et al. 1998 in a similar case of OA, but not with the performance of other OA cases (e.g., Beauvois 1982; Riddoch and Humphreys 1987), who were impaired on such task. Finally, naming of color patches was severely impaired, with spared comprehension of color names (name-to-color matching) and preserved object-color knowledge: this pattern of performance is in double dissociation with that observed in Luzzatti and Davidoff (1994), whose patient suffered from impaired retrieval of object-color knowledge with preserved color naming, and also confirms a relatively preserved access to stored visual knowledge of objects from the phonological input lexicon.

### Lesion-based structural disconnectome study and lesion localization in the corpus callosum

The aim of the present section is to quantify the extent to which brain regions and white-matter tracts are disconnected from the lesion site. Particular emphasis is placed on the analysis on the involvement of the callosal splenium as, according to Schnider and colleagues (1994), the greater the splenial disconnection, the more compatible the ensuing clinical outcome with OA rather than AVA. This was achieved by applying a structural disconnectome method to AA’s lesion (see Hajhajate et al. 2022; for a similar approach). Registration to the standard Montreal Neurological Institute (MNI) template of the skull-stripped T1-weighted structural MRI of the patient was performed using BCBtoolkit (Foulon et al. 2018) with the “classical” masking procedure to weigh the normalization with reference to the brain rather than non-brain tissue or lesions (Brett et al. 2001). The disconnectome map was then calculated using the patient’s lesion (Bonandrini et al. 2020). The disconnectome approach builds on diffusion-weighted images of healthy controls to track the fibers passing through the location of a lesion. In other words, based on structural connectivity in healthy controls, this method estimates the probability - from 0 to 1 - of each volume unit in the brain (voxel) of being disconnected from the lesion site (Thiebaut de Schotten et al. 2015). To do so, the lesion normalized to the MNI152 space is registered to each healthy control native space and used as a seed for tractography (estimated as in Thiebaut de Schotten et al. 2011) in Trackvis (Wang et al. 2007). Each tractography from each control subject is then converted into a visitation map, binarized and back-transformed in the MNI space. Finally, a percentage overlap map is produced by summing, for each voxel of the MNI space, the normalized visitation maps of the healthy subjects. It is worth noting that the disconnectome approach identifies (given a lesioned brain area A) a set of areas (each one can be labelled B) whose connections with the lesioned brain area A are interrupted. All other connections of each brain area B with other areas (each one can be labelled C) other than area A are spared.

Differently from Bonandrini et al. (2020), in which the disconnectome analysis was based on a reference sample of 10 healthy controls, in this analysis we used diffusion weighted images from 100 subjects of the package X (1 mm) available at https://storage.googleapis.com/bcblabweb/open_data.html (the list of track files is reported in the Supplementary Materials). The tracts involved in the disconnection with the lesion site were identified through computation of the voxels of overlap between the disconnectome map (thresholded at a probability of disconnection greater than 0.5) and of the masks from Rojkova and colleagues (2016). The disconnected brain regions were identified using the same approach, but with masks extracted from the HarvardOxford anatomical template. The same template was adopted to extract voxels of overlap with the normalized lesion. The disconnectome and lesion maps were eventually plotted through the FSLeyes software (https://fsl.fmrib.ox.ac.uk/fsl/fslwiki/FSLeyes). The normalized lesion was also plotted on the 2-dimensional medial sagittal plane. The map was masked to show the corpus callosum (through the callosal ROI derived from the JHU atlas), the anterior commissure (spherical ROI of 3 voxels radius centered on the coordinates x = 0, y = 2, z= -5 manually identified on the MNI152 template) and posterior commissure (spherical ROI of 3 voxels radius centered on the coordinates x = 0, y=-26, z= -1 manually identified on the MNI152 template).

**Fig. 5:**
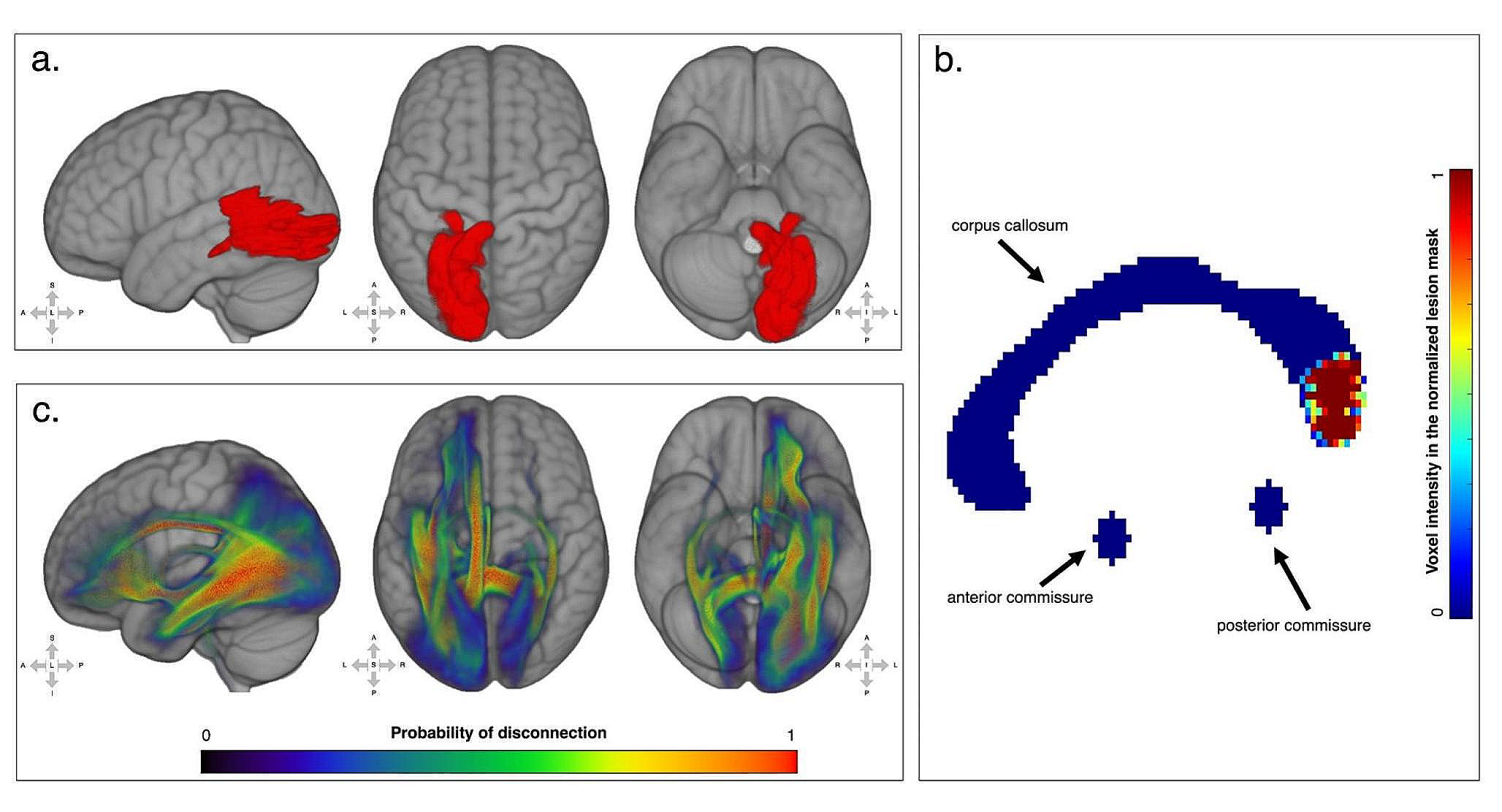
(**a**) 3D rendering of the lesion after normalization to the standard MNI template; (**b**) 2D plot of the lesion at the level of the medial sagittal plane. Fluctuations in voxel intensity at the boundary of the lesion map are a byproduct of spatial normalization; (**c**) 3D rendering of the disconnectome map based on the patient’s lesion and structural connectivity estimates based on 100 healthy controls. For the original MRI scan see Bonandrini et al. (2020) (colors online and in PDF)

*Results and interim discussion*. The lesion mainly involved posterior LH regions (Fig. 5a, Supplementary Table S1), namely the occipital pole, the lingual gyrus, the intracalcarine cortex, the lateral occipital gyrus, the fusiform and parahippocampal gyri, the posterior cingulum and the precuneus. The lesion also involved middle-inferior splenial fibers (Fig. 5b). The disconnectome analysis (Fig. 5c, Supplementary Tables S2 and S3) demonstrated the disconnection from the lesion site of the splenial fibers, together with a set of white matter tracts: the cingulum, the inferior fronto-occipital fasciculus, the optic radiations, the fornix, the long and posterior segments of the arcuate fasciculus, the superior and inferior longitudinal fasciculi.

## General discussion

We described the case of patient AA, who - after a stroke in the territory of the left posterior cerebral artery, displayed a modality-specific deficit in naming line drawings and real objects from sight, but better tactile naming and spared naming to definition. The naming deficit specific for objects presented in the visual modality emerged in AA is – *prima facie* – compatible with both OA and AVA. Although the distinction between AVA and OA may be challenging and some overlapping features may emerge in a same patient, differential diagnosis has been proposed based on: (a) the possibility to access semantic representations from visual input (which should be possible in OA, impaired in AVA; Gerlach and Robotham 2021); (b) the extent of the callosal damage (i.e., extensive splenial involvement and a disconnection of left ventral occipito-temporal areas from right occipital regions, which is more compatible with OA than AVA; Schnider et al., 1994).

As far as the lesion is concerned, the anatomical analysis demonstrated that in AA it encompassed the most posterior (and medial) LH cortical regions, involving the occipital pole, the lingual gyrus, the intracalcarine cortex, the lateral occipital gyrus, the fusiform and parahippocampal gyri, the posterior cingulum and the precuneus. In line with a previous analysis on a more limited reference sample of healthy controls (Bonandrini et al. 2020), we conducted a lesion-based structural disconnectome analysis, which allows to estimate, based on structural connectivity in healthy controls, the probability - from 0 to 1 - of each volume unit in the brain (voxel) of being disconnected from the lesion site (Thiebaut de Schotten et al. 2015). The present disconnectome analysis brings evidence for the critical disconnection of the lesion site (largely involving left ventral occipito-temporal areas) from the RH occipital lobe and to LH language areas. Furthermore, voxels in the middle-inferior callosal splenial fibers, together with a set of white-matter tracts (bilaterally, but mostly in the LH), as the cingulum, the inferior fronto-occipital fasciculus, the optic radiations, the fornix, the long and posterior segments of the arcuate fasciculus turned out to be disconnected from the lesion site (Fig. 5, Supplementary Table S2 and S3). This provides an anatomical account for the visual-verbal disconnection that defines OA: the lesion interrupts the flow of visual information from the RH to the LH occipital cortices, as well as that from LH visual cortices to LH language areas.

From a cognitive perspective, through a series of tasks addressing each step of visual processing, behavioral data demonstrated that AA was able to access structural description knowledge of objects from visual stimuli, correctly distinguishing pictures of real animals from chimeric images. Gerlach and Robotham (2021) proposed a diagnostic flowchart for the assessment of visual agnosia: AVA would be supported by the impaired ability to associate semantically-related line drawings. Conversely, such ability would be spared in OA (Gerlach and Robotham 2021). AA scored poorly in both picture-to-picture matching tasks. However, a poorly compensated right hemianopia and possibly spatial neglect may have affected the processing of stimuli, due to the patient’s tendency to preferentially select left-hand options. In addition, it is worth mentioning that performance on picture-to-picture matching tasks could be hampered by the attempt to use a verbal strategy to solve the visual task, a process that is compromised in OA. Furthermore, AA’s categorization and object color knowledge abilities were preserved, suggesting a spared access to semantics (see Luzzatti et al. 1998; for similar results).

In OA, because of right homonymous hemianopia, visual information is processed by the RH, but it cannot be adequately transferred to the LH for lexical activation, due to the splenial disconnection (Schnider et al. 1994; see Supplementary Figure S1). The disconnectome analysis performed in AA suggests an extensive damage to the splenium, and that the flow of semantic information is most likely to stream leftward through the spared anterior commissure (Fig. 5, see also Fig. 2 for graphical representation of AA’s lesion and disconnection profile with reference to the neurocognitive model). The anterior commissure mediates the connection among inter-hemispheric temporopolar regions (Catani and Thiebaut de Schotten 2008; Mesulam 2023). The inter-hemispheric transfer of information would thus happen more likely after semantic access in RH. It is in the LH that semantic information would trigger activation of the corresponding lexical label. We speculate that in OA the callosal damage would cause impaired or lower, less specific semantic activation among competitors, and consequently naming deficits. Indeed, errors are predominantly semantic, as semantic paraphasias and semantic perseverations. A qualitative analysis of naming errors revealed that AA committed frequent vertical semantic perseverations, namely naming responses biased by those given to previous stimuli (Lhermitte and Beauvois 1973), in line with what described in other cases affected by OA, as well as with the results obtained by Plaut and Shallice (1993) in a simulation study. Differently from lexical perseverations of typical aphasia (Hepner and Nozari 2020), the type of perseveration errors frequently found in OA are considered “a peculiar mingling of semantic errors, perseverations, and descriptions of morphological features of the object” (Beauvois 1982). Explanations in terms of lack of inhibitory processes (Lhermitte and Beauvois 1973), inhibitory connections (Goldenberg and Karlbauer 1998), and damage to “clear-up units” (Plaut and Shallice 1993) have been suggested. To explain typical aphasia perseverations at various levels (e.g., whole words, isolated semantic features, phonemes), Cohen and Dehane (1998) proposed the residual activation model, according to which deafferentation causes persistent activation of information that results in perseverations.

As compared to AA’s difficulties in confrontation naming, in tasks requiring the matching between a phonological entry and visual ones, as in the visual semantic categorization task, providing verbal category labels, AA performed flawlessly. In a similar vein, it is fruitful to analyze the relatively preserved (although overall suboptimal) abilities on the word-to-picture matching tasks. In this tasks, patients classified as having OA usually show a spared or minimally impaired performance (Barca et al. 2009; Chanoine et al. 1998; Coslett and Saffran 1989; Coslett and Saffran 1992; Endo, 1996; Goldenberg and Karlbauer 1998; Kwon et al., 2006; Lhermitte and Beauvois 1973; Lindeboom and Savinkels 1986; Marsh and Hillis 2005; Pena-Casanova et al., 1985; Rapcsak et al. 1987; Riddoch and Humphreys 1987), suggesting the deficit might be unidirectional (Goldenberg and Karlbauer 1998; Luzzatti et al. 1998; but see Lhermitte and Beauvois 1973). Word-to-picture matching requires coupling between an auditorily-presented lexical label and a target picture presented among other visual and/or semantic distractors. For AA to perform the word-to-picture matching task, visual input information has to pass the RH early visual processing and access visual-specific and possibly amodal semantic representations in the RH. Information would then get transferred to the LH (most likely through spared anterior connections) to be compared with the input auditory phonological string. While in the case of confrontation naming, semantic activation is only based on the non-verbal semantic information extracted in the RH, in the case of word-to-picture matching task such semantic activation can be summed to that induced by the input available from the auditory modality. We speculate that this would produce more specific semantic activation, which would in turn result in the accurate selection of the visual target among a set of competitors.

The present interpretation of OA suggests that objects presented visually activate semantic representations in the RH and such semantic activation would be transferred to the LH. Yet, the resulting semantic activation would be insufficient to trigger activation of the correct lexical label. This would be because no vision-based semantic activation occurs in the LH due to the lesion impairing the LH visual cortex and the splenial interruption of the connections with the RH occipital areas. Whenever in this system additional information is added so as to produce increased semantic activation (for instance by presenting the label of the target word auditorily such as in the word-to-picture matching task or in the categorization task in which semantic labels are provided), the amount of semantic activation becomes sufficient to induce the lexical activation of the target item. A similar mechanism would explain AA’s difficulties in pantomiming objects. As shown in Fig. 2, the impaired semantic information transferred to the LH, would not activate the corresponding ideational gesture representation, in the same vein as for the phonological output lexicon, although AA also showed ideomotor apraxia for meaningless gestures.

Still the issue remains of how damage to the posterior cerebral artery territory can sometimes cause OA, and sometimes AVA. One possibility is that AVA is likely to arise when lesions in the left hemisphere disconnect posterior occipital areas from more anterior areas in the temporal lobe (Carlesimo et al., 1998; Gerlach and Robotham 2021). Schnider et al. (1994) suggested that the core difference is between the extension of splenial disconnection: a complete disconnection would enable the information processing in the RH, giving rise to symptoms consistent with OA, whereas incomplete splenial disconnection would allow LH information processing. In this regard the deafferentation of other LH white matter fasciculi could play a prominent role (De Renzi 1996; De Renzi and Saetti 1997). In particular, the disconnection of the inferior longitudinal fasciculus could impair the transfer of visual information (Catani 2003) from posterior temporal and occipital lateral cortices to the anterior temporal semantic storage areas, which allow for visual object identification (Catani and Mesulam 2008). Deafferentation of the inferior fronto-occipital fasciculus could further contribute to visuo-verbal disconnection (see for instance the functions of this tract as proposed by Catani and Thiebaut de Schotten 2008).

The present study suggests that in OA, while splenial disconnection impedes interhemispheric communication of early visual information, semantic information could be transferred from RH to LH, possibly through the anterior commissure. Future disconnectome-based studies may clarify the role of a disconnection between temporal lobes via anterior commissure in preventing access to semantic representations in AVA.

In conclusion, the present report suggests splenial disconnection and some spared semantic access from visual input in a patient showing a deficit in visual object naming. The available clinical and neuroimaging data support an interpretation of the case that is more compatible with OA than with AVA. This study constitutes, to the best of our knowledge, the first attempt to combine a cognitive and neuropsychological approach to the differential diagnosis of AVA/OA with a disconnectome analysis of the lesion profile. The study underlines the importance to explore white-matter disconnection(s) in the emergence of rare neuropsychological symptom sets.

## Electronic supplementary material

Below is the link to the electronic supplementary material.

Supplementary Material 1

## Data Availability

Data will be made available on reasonable request.

## Footnotes

1 De Renzi (1999) claimed that premorbid variability of RH lexical-semantic competence itself could underlie the different clinical outcomes, based on the different degree to which the RH can compensate for the LH’s lack of contribution to visual semantic processing. Although plausible, empirical support for this interpretation would rely on the availability of premorbid data. As such, this hypothesis proves difficult to demonstrate.

## References

[CR1] Albert ML (1973) A simple test of visual neglect. Neurology 23:658–664. 10.1212/WNL.23.6.658 4736313 10.1212/WNL.23.6.658

[CR2] Banco E, Veronelli L, Briguglio M, Luzzatti C, Vallar G (2023) The Semantic Association Test (SAT): normative data from healthy Italian participants and a validation study in aphasic patients. Neurol Sci 44(5):1575–1586. 10.1007/s10072-022-06543-5 36572752 10.1007/s10072-022-06543-5

[CR3] Barca L, Cappelli FR, Amicuzi I, Apicella MG, Castelli E, Stortini M (2009) Modality-specific naming impairment after traumatic brain injury (TBI). Brain Inj 23(11):920–929. 10.1080/02699050903283205 20100129 10.1080/02699050903283205

[CR4] Bartolomeo P (2022) Visual objects and their colors. Handb Clin Neurol 187:179–189. 10.1016/B978-0-12-823493-8.00022-5 35964971 10.1016/B978-0-12-823493-8.00022-5

[CR5] Beauvois MF (1982) Optic aphasia: a process of interaction between vision and language. Philos Trans R Soc Lond B Biol Sci 298(1089):35–47. 10.1098/rstb.1982.0070 6125974 10.1098/rstb.1982.0070

[CR6] Binder JR, Mohr JP (1992) The topography of callosal reading pathways: a case-control analysis. Brain 115:1807–1826. 10.1093/brain/115.6.1807 1486462 10.1093/brain/115.6.1807

[CR7] Bonandrini R, Veronelli L, Licciardo D, Caporali A, Judica E, Corbo M, Luzzatti C (2020) Can the right hemisphere read? A behavioral and disconnectome study on implicit reading in a patient with pure alexia. NeuroCase 26(6):321–327. 10.1080/13554794.2020.1830118 33026948 10.1080/13554794.2020.1830118

[CR8] Brett M, Leff AP, Rorden C, Ashburner J (2001) Spatial normalization of brain images with focal lesions using cost function masking. NeuroImage 14(2):486–500. 10.1006/nimg.2001.0845 11467921 10.1006/nimg.2001.0845

[CR9] Campbell R, Manning L (1996) Optic aphasia: a case with spared action naming and associated disorders. Brain Lang 53(2):183–221. 10.1006/brln.1996.0044 8726533 10.1006/brln.1996.0044

[CR10] Capitani E, Laiacona M (1997) Composite neuropsychological batteries and demographic correction: standardization based on equivalent scores, with a review of published data. J Clin Exp Neuropsychol 19(6):795–809. 10.1080/01688639708403761 9524875 10.1080/01688639708403761

[CR87] Carlesimo GA, Casadio P, Sabbadini M, Caltagirone C (1998) Associative visual agnosia resulting from a disconnection between intact visual memory and semantic systems. Cortex 34(4):563–576. 10.1016/S0010-9452(08)70514-8 10.1016/s0010-9452(08)70514-8 9800090

[CR11] Catani M (2003) Occipito-temporal connections in the human brain. Brain 126(9):2093–2107. 10.1093/brain/awg203 12821517 10.1093/brain/awg203

[CR13] Catani M, Thiebaut de Schotten M (2008) A diffusion tensor imaging tractography atlas for virtual in vivo dissections. Cortex 44(8):1105–1132. 10.1016/j.cortex.2008.05.004 18619589 10.1016/j.cortex.2008.05.004

[CR12] Catani M, Mesulam M (2008) The arcuate fasciculus and the disconnection theme in language and aphasia: history and current state. Cortex 44(8):953–961. 10.1016/j.cortex.2008.04.002 18614162 10.1016/j.cortex.2008.04.002 PMC2740371

[CR14] Catricalà E, Della Rosa PA, Ginex V, Mussetti Z, Plebani V, Cappa SF (2013) An Italian battery for the assessment of semantic memory disorders. Neurol Sci 34(6):985–993. 10.1007/s10072-012-1181-z 22960873 10.1007/s10072-012-1181-z

[CR15] Chanoine V, Ferreira CT, Demonet JF, Nespoulous JL, Poncet M (1998) Optic aphasia with pure alexia: a mild form of visual associative agnosia? A case study. Cortex 34(3):437–448. 10.1016/S0010-9452(08)70766-4 9669108 10.1016/S0010-9452(08)70766-4

[CR16] Cohen L, Dehaene S (1998) Competition between past and present. Assessment and interpretation of verbal perseverations. Brain 121(9):1641–1659. 10.1093/brain/121.9.1641 9762954 10.1093/brain/121.9.1641

[CR17] Colombo A, De Renzi E, Faglioni P (1976) The occurrence of visual neglect in patients with unilateral cerebral disease. Cortex 12:221–231. 10.1016/S0010-9452(76)80003-2 1000990 10.1016/S0010-9452(76)80003-2

[CR18] Coslett HB, Saffran EM (1989) Preserved object recognition and reading comprehension in optic aphasia. Brain 112:1091–1110. 10.1093/brain/112.4.1091 2775993 10.1093/brain/112.4.1091

[CR19] Coslett HB, Saffran EM (1992) Optic aphasia and the right hemisphere: a replication and extension. Brain Lang 43(1):148–161. 10.1016/0093-934X(92)90026-B 1643508 10.1016/0093-934X(92)90026-B

[CR20] Damasio AR, Damasio H (1983) The anatomic basis of pure alexia. Neurology 33(12):1573–1583. 10.1212/WNL.33.12.1573 6685830 10.1212/WNL.33.12.1573

[CR22] De Renzi E (1996) Le agnosie visive. In: Denes G, Pizzamiglio L (eds) Manuale di Neuropsicologia. Normalità e Patologia dei Processi Cognitivi, 2nd edn. Zanichelli, Bologna, pp 497–541, Engl. trans. by, De Renzi E (1999) Visual agnosias. In: Denes G, Pizzamiglio L (eds) Handbook of Clinical and Experimental Neuropsychology, Psychology Press, London

[CR23] De Renzi E, Saetti MC (1997) Associative agnosia and optic aphasia: qualitative or quantitative difference? Cortex 33(1):115–130. 10.1016/S0010-9452(97)80008-1 9088725 10.1016/S0010-9452(97)80008-1

[CR21] De Renzi E, Zambolin A, Crisi G (1987) The pattern of neuropsychological impairment associated with left posterior cerebral artery infarcts. Brain 110:1099–1116. 10.1093/brain/110.5.1099 3676694 10.1093/brain/110.5.1099

[CR24] Della Sala S, Laiacona M, Trivelli C, Spinnler H (1995) Poppelreuter-Ghent’s overlapping figures test: its sensitivity to age, and its clinical use. Arch Clin Neuropsychol 10(6):511–534. 10.1093/arclin/10.6.511 14588906 10.1093/arclin/10.6.511

[CR85] De Renzi E, Motti F, Nichelli P (1980) Imitating gestures. A quantitative approach to ideomotor apraxia. Arch Neurol 37(1):6–10. 10.1001/archneur.1980.00500500036003 10.1001/archneur.1980.00500500036003 7350907

[CR26] Endo K, Makishita H, Yanagisawa N, Sugishita M (1996) Modality specific naming and gesture disturbances: a case with optic aphasia, bilateral tactile aphasia, optic apraxia and tactile apraxia. Cortex 32(1):3–28. 10.1016/s0010-9452(96)80014-1 8697751 10.1016/s0010-9452(96)80014-1

[CR27] Farah M (1990) Visual Agnosia. MIT Press, Cambridge, MA

[CR28] Feinberg TE, Schindler RJ, Ochoa E, Kwan PC, Farah MJ (1994) Associative visual agnosia and alexia without prosopagnosia. Cortex 30(3):395–411. 10.1016/s0010-9452(13)80337-1 7805382 10.1016/s0010-9452(13)80337-1

[CR29] Foulon C, Cerliani L, Kinkingnehun S, Levy R, Rosso C, Urbanski M, Volle E, Thiebaut de Schotten M (2018) Advanced lesion symptom mapping analyses and implementation as BCBtoolkit. Gigascience 7(3):giy004. 10.1093/gigascience/giy004 29432527 10.1093/gigascience/giy004 PMC5863218

[CR30] Freud S (1891) Zur Auffassung Der Aphasien. Franz Deuticke, Leipzig and Wien

[CR31] Freund CS (1889) Über optische Aphasie und Seelenblindheit. Archiv f. Psychiatrie 20(1):276–297. 10.1007/BF02041542, Engl. trans. by, Beaton A, Davidoff J, Erstfeld U (1991) On optic aphasia and visual agnosia. Cogn Neuropsychol 8:21–38. 10.1080/02643299108253365

[CR32] Gainotti G, Marra C (2011) Differential contribution of right and left temporo-occipital and anterior temporal lesions to face recognition disorders. Front Hum Neurosci 1:5:55. 10.3389/fnhum.2011.00055PMID: 21687793; PMCID: PMC3108284 10.3389/fnhum.2011.00055 PMC3108284 21687793

[CR33] Gamboz N, Coluccia E, Iavarone A, Brandimonte MA (2009) Normative data for the pyramids and Palm Trees Test in the elderly Italian population. Neurol Sci 30(6):453–458. 10.1007/s10072-009-0130-y 19768374 10.1007/s10072-009-0130-y

[CR34] Gauthier L, Dehaut F, Joanette Y (1989) The bells Test: a quantitative and qualitative test for visual neglect. Int J Clin Neuropsychol 11:49–54

[CR35] Gerlach C, Robotham RJ (2021) Object recognition and visual object agnosia. Handb Clin Neurol 178:155–173. 10.1016/B978-0-12-821377-3.00008-8 33832675 10.1016/B978-0-12-821377-3.00008-8

[CR36] Geschwind N (1965) Disconnexion syndromes in animals and man. Brain 88(3):585–644. 10.1093/brain/88.3.585 5318824 10.1093/brain/88.2.237;10193/brain/88.3.585

[CR37] Goldenberg G, Karlbauer F (1998) The more you know the less you can tell: inhibitory effects of visuo-semantic activation on modality specific visual misnaming. Cortex 34(4):471–491. 10.1016/S0010-9452(08)70509-4 9800085 10.1016/S0010-9452(08)70509-4

[CR38] Goldstein K (1906) Zur Frage Der Amnestischen Aphasie und ihrer Abgrenzung gegenüber Der Transcorticalen Und Glossopsychischen Aphasie. Arch Psychiatr Nervenkrankh 41(3):911–950 10.1007/BF02058005

[CR39] Hajhajate D, Kaufmann BC, Liu J, Siuda-Krzywicka K, Bartolomeo P (2022) The connectional anatomy of visual mental imagery: evidence from a patient with left occipito-temporal damage. Brain Struct Funct 227(9):3075–3083. 10.1007/s00429-022-02505-x 35622159 10.1007/s00429-022-02505-x

[CR40] Hepner CR, Nozari N (2020) The dual origin of lexical perseverations in aphasia: residual activation and incremental learning. Neuropsychologia 147:107603. 10.1016/j.neuropsychologia.2020.107603 32877655 10.1016/j.neuropsychologia.2020.107603

[CR41] Hillis AE, Caramazza A (1995) Cognitive and neural mechanisms underlying visual and semantic processing: implications from optic aphasia. J Cogn Neurosci 7(4):457–478. 10.1162/jocn.1995.7.4.457 23961905 10.1162/jocn.1995.7.4.457

[CR42] Howard D, Patterson KE (1992) The pyramids and Palm Trees Test. Valley Test Company, Bury St Edmonds

[CR43] Huber W, Poeck K, Willmes K (1984) The Aachen Aphasia Test. In: Rose FC (ed) Advances in Neurology. Progress in Aphasiology, vol 42. Raven, New York, pp 291–303 6209953

[CR44] Humphreys GW, Riddoch JM (1993) Birmingham Object Recognition Battery. Psychology Press. 10.1037/t13731-000

[CR45] Kleist K (1934) Gehirnpathologie. Handbuch Der ärztlichen Erfahrungen Im Weltkriege 1914–1918, vol 4. Barth, Leipzig

[CR46] Kwon M, Lee JH (2006) Optic aphasia: a case study. J Clin Neurol 2(4):258–261. 10.3988/jcn.2006.2.4.258 20396529 10.3988/jcn.2006.2.4.258 PMC2854976

[CR47] Leek C, Rapp B, Caramazza A (1994) Accessing semantics from vision: the case study of a patient with a visual modality-specific naming impairment. Brain Lang 47:323–325

[CR48] Lhermitte F, Beauvois MF (1973) A visual-speech disconnexion syndrome: report of a case with optic aphasia, agnosic alexia and colour agnosia. Brain 96(4):695–714. 10.1093/brain/96.4.695 4773861 10.1093/brain/96.4.695

[CR49] Lindeboom J, Savinkels JA (1986) Interhemispheric communication in a case of total visuo-verbal disconnection. Neuropsychologia 24:781–792. 10.1016/0028-3932(86)90077-1 3808286 10.1016/0028-3932(86)90077-1

[CR50] Lissauer H (1890) Ein Fall Von Seelenblindheit nebst einem Beitrage Zur Theorie Derselben. Arch Psychiatr Nervenkrankh 21(2):222–270 10.1007/BF02226765

[CR51] Luzzatti C (2003) Optic aphasia and pure alexia: contribution of callosal disconnection syndromes to the study of lexical and semantic representation in the right hemisphere. In: Zaidel E, Iacoboni M (eds) The parallel brain: the cognitive neuroscience of the Corpus Callosum. MIT Press, Cambridge, MA, pp 479–499

[CR52] Luzzatti C, Davidoff J (1994) Impaired retrieval of object-colour knowledge with preserved colour naming. Neuropsychologia 32:933–950. 10.1016/0028-3932(94)90044-2 7969868 10.1016/0028-3932(94)90044-2

[CR55] Luzzatti C, Rumiati RI, Ghirardi G (1998) A functional model of visuo-verbal disconnection and the neuroanatomical constraints of optic aphasia. NeuroCase 4(1):71–87. 10.1080/13554799808410609 10.1080/13554799808410609

[CR54] Luzzatti C, Mauri I, Castiglioni S, Zuffi M, Spartà C, Somalvico F, Franceschi M (2020) Evaluating Semantic Knowledge through a Semantic Association Task in individuals with dementia. Am J Alzheimers Dis Other Demen 35:1533317520917294. 10.1177/1533317520917294 32308008 10.1177/1533317520917294 PMC10623912

[CR53] Luzzatti C, De Bleser R, Scola I, Frustaci M, Willmes K (2023) Update on the psychometric properties for the Italian version of the Aachen Aphasia Test (IT-AAT). Aphasiology 37:658–695. 10.1080/02687038.2022.2037501 10.1080/02687038.2022.2037501

[CR56] Luzzatti C, Willmes K, De Bleser R (2024) Aachener Aphasie Test: Versione Italiana, 3rd edn. Hogrefe Italia, Firenze. (in press)

[CR57] Manning L, Campbell R (1992) Optic aphasia with spared action naming: a description and possible loci of impairment. Neuropsychologia 30(6):587–592. 10.1016/0028-3932(92)90061-P 1379351 10.1016/0028-3932(92)90061-P

[CR58] Marr D (1980) Visual information processing: the structure and creation of visual representations. Philos Trans R Soc Lond B Biol Sci 290(1038):199–218. 10.1098/rstb.1980.0091 6106238 10.1098/rstb.1980.0091

[CR59] Marsh EB, Hillis AE (2005) Cognitive and neural mechanisms underlying reading and naming: evidence from letter-by-letter reading and optic aphasia. NeuroCase 11(5):325–337. 10.1080/13554790591006320 16251134 10.1080/13554790591006320

[CR60] McCarthy RA, Warrington EK (1986) Visual associative agnosia: a clinico-anatomical study of a single case. J Neurol Neurosurg Psychiatry 49(11):1233–1240. 10.1136/jnnp.49.11.1233 3794729 10.1136/jnnp.49.11.1233 PMC1029070

[CR61] Mesulam MM (2023) Temporopolar regions of the human brain. Brain 146(1):20–41. 10.1093/brain/awac339 36331542 10.1093/brain/awac339 PMC10060717

[CR62] Miceli G, Laudanna A, Burani C, Capasso R (1994) Batteria per l’Analisi dei Deficit Afasici (BADA). CEPSAG, Università Cattolica del Sacro Cuore, Rome

[CR63] Mohr JP, Binder JR (2011) Posterior cerebral artery disease. In: Mohr JP, Wolf PA, Grotta JC, Moskowitz MA, Mayberg MR, von Kummer RBT (ed) Stroke, 5th edn. WB Saunders (Elsevier), Philadelphia, PA, Chap. 25; 425–445

[CR64] Novelli G, Papagno C, Capitani E, Laiacona M, Vallar G, Cappa SF (1986) Tre test clinici di ricerca e produzione lessicale. Archivio Di Psicologia Neurologia E Psichiatria m47(4):477–506 Taratura su soggetti normali

[CR86] Patterson K, Nestor PJ, Rogers TT (2007) Where do you know what you know? The representation of semantic knowledge in the human brain. Nat Rev Neurosci 8(12):976–87. 10.1038/nrn2277 10.1038/nrn2277 18026167

[CR65] Peña-Casanova J, Roig-Rovira T, Bermudez A, Tolosa-Sarro E (1985) Optic aphasia, optic apraxia, and loss of dreaming. Brain Lang 26(1):63–71. 10.1016/0093-934X(85)90028-8 2413956 10.1016/0093-934X(85)90028-8

[CR66] Plaut DC, Shallice T (1993) Perseverative and semantic influences on visual object naming errors in optic aphasia: a connectionist account. J Cogn Neurosci 5(1):89–117. 10.1162/jocn.1993.5.1.89 23972122 10.1162/jocn.1993.5.1.89

[CR67] Ptak R, Lazeyras F, Di Pietro M, Schnider A, Simon SR (2014) Visual object agnosia is associated with a breakdown of object-selective responses in the lateral occipital cortex. Neuropsychologia 60:10–20. 10.1016/j.neuropsychologia.2014.05.009 24863251 10.1016/j.neuropsychologia.2014.05.009

[CR68] Rapcsak SZ, Gonzalez Rothi LJ, Heilman KM (1987) Phonological alexia with optic and tactile anomia: a neuropsychological and anatomical study. Brain Lang 31(1):109–121. 10.1016/0093-934X(87)90063-0 3580836 10.1016/0093-934X(87)90063-0

[CR69] Ratcliff TG, Newcombe F (1982) Object recognition: some deductions from the clinical evidence. In: Ellis AW (ed) Normality and Pathology in Cognitive functions. Academic, New York

[CR70] Raymer AM, Greenwald ML, RichardsonM, Rothi LJG, Heilman KM (1997) The right hemisphere and optic aphasia/optic apraxia. NeuroCase 3(3):173–183. 10.1080/13554799708404052 10.1080/13554799708404052

[CR71] Riddoch MJ, Humphreys GW (1987) Visual object processing in optic aphasia: a case of semantic access agnosia. Cogn Neuropsych 4(2):131–185. 10.1080/02643298708252038 10.1080/02643298708252038

[CR72] Rojkova K, Volle E, Urbanski M, Humbert F, Dell’Acqua F, Thiebaut de Schotten M (2016) Atlasing the frontal lobe connections and their variability due to age and education: a spherical deconvolution tractography study. Brain Struct Funct 221:1751–1766. 10.1007/s00429-015-1001-3 25682261 10.1007/s00429-015-1001-3

[CR73] Schnider A, Benson DF, Scharre DW (1994) Visual agnosia and optic aphasia: are they anatomically distinct? Cortex 30(3):445–457. 10.1016/S0010-9452(13)80340-1 7805385 10.1016/S0010-9452(13)80340-1

[CR74] Schweiger A, Zaidel E, Field T, Dobkin B (1989) Right hemisphere contribution to lexical access in an aphasic with deep dyslexia. Brain Lang 37(1):73–89. 10.1016/0093-934X(89)90102-8 2752276 10.1016/0093-934X(89)90102-8

[CR75] Semenza C, Luzzatti C (2019) Disturbi Lessicali Nell’afasia. In: Denes G, Pizzamiglio L, Guariglia C, Cappa SF, Grossi D, Luzzatti C (eds) Manuale Di Neuropsicologia. Zanichelli, Bologna

[CR76] Sitton M, Mozer MC, Farah MJ (2000) Superadditive effects of multiple lesions in a connectionist architecture: implications for the neuropsychology of optic aphasia. Psychol Rev 107(4):709. 10.1037/0033-295X.107.4.709 11089404 10.1037/0033-295X.107.4.709

[CR77] Siuda-Krzywicka K, Witzel C, Chabani E, Taga M, Coste C, Cools N, Ferrieux S, Cohen L, Seidel Malkinson T, Bartolomeo P (2019) Color Categorization Independent of Color naming. Cel Rep 28:2471–2479e5. 10.1016/j.celrep.2019.08.003 10.1016/j.celrep.2019.08.003 31484060

[CR78] Spinnler H, Tognoni G (1987) Standardizzazione E taratura italiana di test neuropsicologici. [Italian normative values and standardization of neuropsychological tests]. Ital J Neurol Sci 6(Suppl 8):1–120 3330072

[CR79] Thiebaut de Schotten M, Dell’Acqua F, Forkel S, Simmons A, Vergani F, Murphy DG, Catani M (2011) A lateralized brain network for visuo-spatial attention. Nat Neurosci 14(10):1245–1246. 10.1038/nn.2905 21926985 10.1038/nn.2905

[CR80] Thiebaut de Schotten M, Dell’Acqua F, Ratiu P, Leslie A, Howells H, Cabanis E, Iba-Zizen MT, Plaisant O, Simmons A, Dronkers NF, Corkin S, Catani M (2015) From Phineas Gage and Monsieur Leborgne to H. M : Revisiting Disconnection Syndr Cereb Cortex 25(12):4812–4827. 10.1093/cercor/bhv173 10.1093/cercor/bhv173 PMC4635921 26271113

[CR81] Vallar G, Rusconi ML, Fontana S, Musicco M (1994) Tre test di esplorazione visuo-spaziale: taratura su 212 soggetti normali. Arch Psicol Neurol Psichiatr 55:827–841

[CR82] Wang R, Benner T, Sorensen AG, Wedeen VJ (2007) Diffusion toolkit: a software package for diffusion imaging data processing and tractography. Proc Intl Soc Mag Reson Med 15:3720

[CR83] Wolff G (1904) Klinische Und Kritische Beiträge Zur Lehre Von Den Sprachstörungen. Veit, Leipzig

[CR84] Zaidel E (1991) Language functions in the two hemispheres following complete cerebral commissurotomy and hemispherectomy. In: Boiler F, Graffman J (eds) Handbook of Neuropsychology. Elsevier, Amsterdam, pp 115–150

