# Supplementary material for: Clinical and structural disconnectome evaluation in a case of optic aphasia
Source: Brain Struct Funct. 2024 Jun 25;229(7):1641–54. doi: 10.1007/s00429-024-02818-z (PMC11374911; doi:10.1007/s00429-024-02818-z)
Supplement: Supplementary file 1 — Supplementary Material 1 [file 429_2024_2818_MOESM1_ESM.pdf]

Clinical and structural disconnectome evaluation in a case of Optic Aphasia

Laura Veronelli[1,2,*], Rolando Bonandrini[1], Alessandra Caporali[2], , Daniele Licciardo[1,3], Massimo Corbo[2], Claudio Luzzatti[1]

[1] Department of Psychology, University of Milano-Bicocca and Milan Center for Neuroscience, Milan, Italy.

[2] Department of Neurorehabilitation Sciences, Casa di Cura IGEA, Milan, Italy.

[3] Department of Medicine and Surgery, University of Milano-Bicocca, Monza, Italy.

SUPPLEMENTARY MATERIALS

Correspondence to:

Laura Veronelli, Ph.D.

University of Milano-Bicocca, Department of Psychology

Piazza dell'Ateneo Nuovo 1, 20126 Milan, Italy

tel: +39 0264483704

e-mail: laura.veronelli@unimib.it

Supplementary Table S1: Regions of the HarvardOxford template showing at least one voxel of overlap with the normalized lesion.

Region	***Number of overlapping voxels***
Lingual Gyrus L	11021
Occipital Fusiform Gyrus L	7376
Occipital Pole L	6360
Intracalcarine Cortex L	5139
Precuneous Cortex L	4045
Cingulate Gyrus, posterior division L	3683
Temporal Occipital Fusiform Cortex L	2801
Parahippocampal Gyrus, posterior division L	1496
Lateral Occipital Cortex, inferior division L	1173
Supracalcarine Cortex L	361
Cingulate Gyrus, posterior division R	255
Cuneal Cortex L	141
Temporal Fusiform Cortex, posterior division L	88
Lateral Occipital Cortex, superior division L	10
Inferior Temporal Gyrus, temporooccipital part L	6
Intracalcarine Cortex R	3
Lingual Gyrus R	1

L: left; R: right.

Supplementary Table S2: Tracts from Rojkova et al. (2016) showing at least one voxel of disconnection with the lesion site (disconnectome map thresholded at 0.5).

Tract	***Number of voxels disconnected from the lesion site ($p_{disconnection} > 0.5$)***
Corpus Callosum	71333
Inferior Longitudinal L	39008
Inferior Fronto Occipital Fasciculus L	25551
Optic Radiations L	24280
Anterior Commissure	23172
Cingulum L	15643
Fornix	9434
Cingulum Posterior L	9237
Cingulum Anterior L	7874
Inferior Longitudinal R	7114
Fronto Striatal L	6803
Frontal Commissural	5359
Uncinate L	5186
Inferior Fronto Occipital Fasciculus R	5163
Anterior Thalamic Projections L	4767
Pons L	4634
Optic Radiations R	3969
Superior Longitudinal Fasciculus II L	3314
Superior Longitudinal Fasciculus I L	3313
Cingulum Posterior R	2513
Cingulum R	2467
Frontal Orbito Polar L	2256
Superior Longitudinal Fasciculus III L	2220
Cingulum Anterior R	1707
Arcuate Long Segment L	1247
Cortico Spinal L	873
Superior Londgitudinal Fasciculus I R	784
Superior Londgitudinal Fasciculus II R	724
Frontal Aslant Tract L	557
Fronto Striatal R	260
Arcuate Posterior Segment L	242
Anterior Thalamic Projections R	228
Uncinate R	185
Frontal Superior Longitudinal L	184
Superior Longitudinal Fasciculus III R	158
Pons R	134
Fronto Insular Tract2 L	96
Fronto Insular Tract3 L	54
Fronto Insular Tract1 L	51
Frontal Inferior Longitudinal L	34

Fronto Marginal Tract L	22
Arcuate Anterior Segment L	17
Handinf U Tract L	15
Fronto Insular Tract4 L	4
Arcuate Posterior Segment R	1

L: left; R: right.

Supplementary Table S3: Regions of the HarvardOxford template showing at least one voxel of disconnection with the lesion site (disconnectome map thresholded at 0.5).

Region	***Number of voxels disconnected from the lesion site ($p_{disconnection} > 0.5$)***
Cingulate Gyrus, posterior division L	5576
Lingual Gyrus L	4609
Precuneous Cortex L	4535
Intracalcarine Cortex L	3708
Precuneous Cortex R	3354
Cingulate Gyrus, posterior division R	2897
Occipital Fusiform Gyrus L	2839
Lateral Occipital Cortex, inferior division L	2360
Parahippocampal Gyrus, posterior division L	2355
Temporal Fusiform Cortex, posterior division L	2116
Temporal Occipital Fusiform Cortex L	1980
Cingulate Gyrus, anterior division L	1864
Cuneal Cortex L	1754
Lateral Occipital Cortex, superior division L	1619
Intracalcarine Cortex R	1610
Parahippocampal Gyrus, anterior division L	1159
Occipital Pole L	1157
Insular Cortex L	1101
Frontal Orbital Cortex L	1079
Lingual Gyrus R	945
Frontal Pole L	791
Inferior Temporal Gyrus, posterior division L	731
Supracalcarine Cortex R	604
Temporal Fusiform Cortex, anterior division L	539
Paracingulate Gyrus L	532
Supracalcarine Cortex L	418
Planum Polare L	365
Inferior Temporal Gyrus, temporooccipital part L	335
Inferior Temporal Gyrus, anterior division L	290
Angular Gyrus L	274
Parahippocampal Gyrus, posterior division R	255
Temporal Fusiform Cortex, posterior division R	223
Middle Temporal Gyrus, posterior division L	216
Superior Temporal Gyrus, posterior division L	171
Superior Temporal Gyrus, anterior division L	158
Temporal Pole L	137
Cuneal Cortex R	130
Middle Temporal Gyrus, temporooccipital part L	114
Subcallosal Cortex L	113
Temporal Occipital Fusiform Cortex R	112

Supramarginal Gyrus, anterior division L	96
Superior Parietal Lobule R	71
Occipital Fusiform Gyrus R	67
Frontal Medial Cortex L	63
Inferior Frontal Gyrus, pars triangularis L	62
Superior Parietal Lobule L	61
Occipital Pole R	60
Middle Temporal Gyrus, anterior division L	58
Planum Temporale L	43
Supramarginal Gyrus, posterior division L	41
Lateral Occipital Cortex, superior division R	39
Frontal Operculum Cortex L	34
Postcentral Gyrus R	29
Parahippocampal Gyrus, anterior division R	19
Lateral Occipital Cortex, inferior division R	19
Supplementary Motor Cortex L	17
Angular Gyrus R	14
Middle Frontal Gyrus L	13
Heschls Gyrus (includes H1 and H2) L	4
Inferior Temporal Gyrus, posterior division R	4
Precentral Gyrus L	4
Parietal Operculum Cortex L	2
Insular Cortex R	1
Superior Frontal Gyrus L	1
Temporal Fusiform Cortex, anterior division R	1

L: left; R: right.

Supplementary Table S4. Anatomical location -according to available literature- of the units included in the neurocognitive model pictured in Figure S1.

Component	***Anatomical location***	***Reference***
Visual analysis	Medial and lateral occipital cortices	Humphreys & Riddoch (2006); Devinsky et al. (2008)
Auditory analysis	STS	Gutschalk et al. (2015)
Phonological input lexicon	Middle-posterior STS	Matchin et al. (2022)
Orthographic input lexicon	Fusiform gyrus	Taylor et al. (2013)
Structural description system	Fusiform gyrus	Ferreira et al. (1998); Vuilleumier et al. (2002)
Verbal semantics	Anterior middle temporal gyrus	Devereaux et al. (2013)
Visual semantics	Ventral temporal cortex	Devereaux et al. (2013)
Semantic hub	Temporal pole	Lambon Ralph et al. (2017)
Phonological output lexicon	Inferior frontal gyrus (pars triangularis)	Taylor et al. (2013)
Ideational gesture representation	Inferior parietal lobule	Niessen et al. (2014)

The reported inter-hemispheric structural connections between the different neurocognitive components were informed by a template-based structural (dis)connectome analysis. This was carried out by means of the BCBToolkit software using the same approach used for the disconnectome analysis on the patient's lesion. For our purposes, this method was used to provide an overview of interhemispheric structural connections passing through the medial sagittal plane. In particular, we used the regions of interest (ROIs) from the HarvardOxford template that are relevant for the model depicted in Figure S1. Structural connectome maps were then averaged for each couple of homologue contralateral ROIs. For each couple of homologue ROIs, finally, the average connection map was plotted on the 2-dimensional medial sagittal plane. The map was masked to show the corpus callosum (through the callosal ROI derived from the JHU atlas), the anterior commissure (spherical ROI of 3 voxels radius centered on the coordinates x=0, y=2, z= -5 manually identified on the MNI152 template) and posterior commissure (spherical ROI of 3 voxels radius centered on the coordinates x=0, y=-26, z= -1 manually identified on the MNI152 template). Figure S2 shows, for each ROI, the probability that a white matter tract coming from that ROI intersects the medial sagittal plane at the level of the corpus callosum, anterior, or posterior commissure.

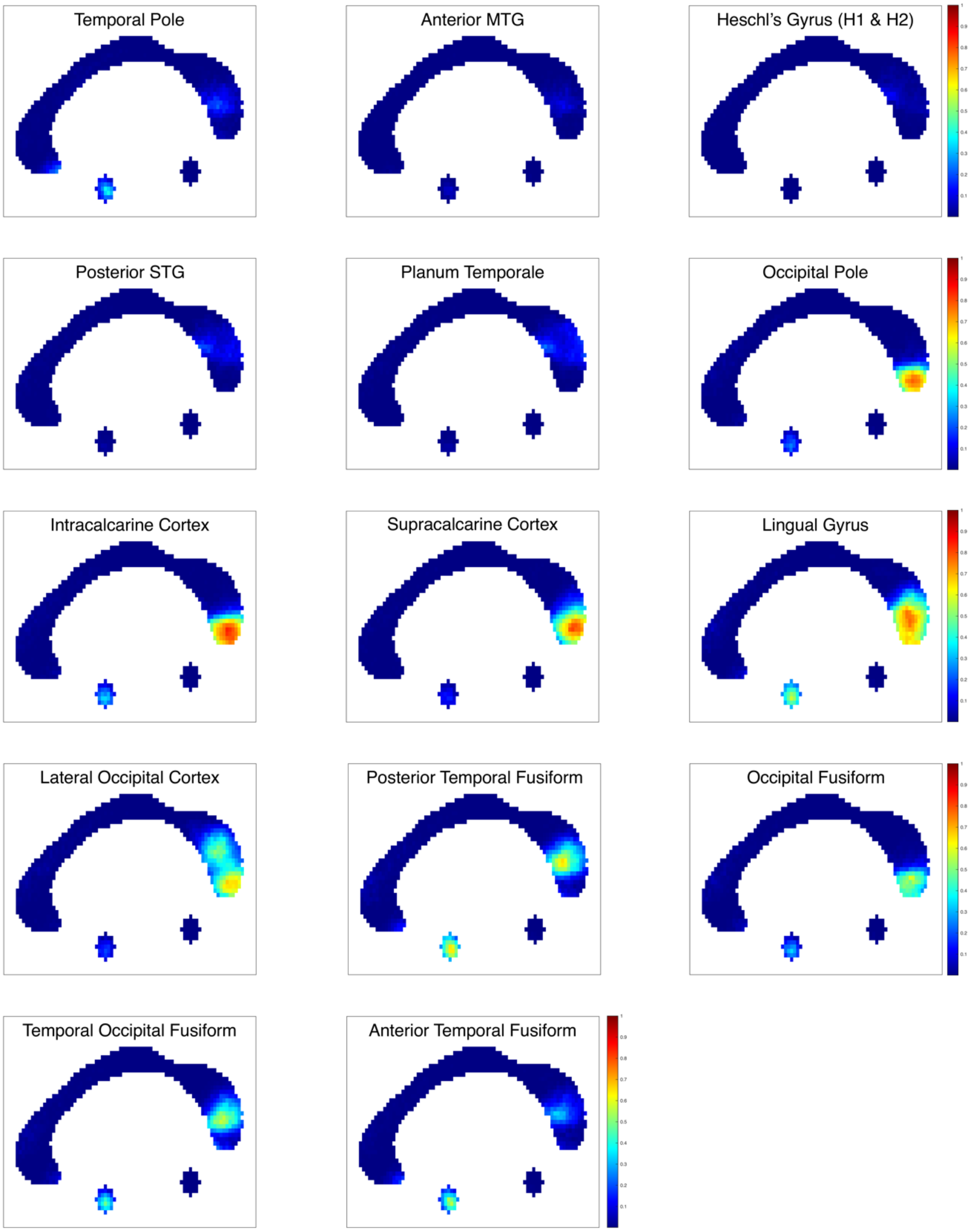

Supplementary Figure S1. Projection of the template-based (dis)connection maps onto the medial sagittal plane. The colormap indicates the probability of structural (dis)connection with the ROI: red= 1, blue=0. STG= superior temporal gyrus, MTG= middle temporal gyrus.

List of input track filenames for structural connectome analysis. Diffusion weighted images from 100 subjects of the package X (1 mm) available at https://storage.googleapis.com/bcblabweb/open_data.html used as reference for the disconnectome analysis.

100610.trk	128935.trk	148133.trk	169747.trk	186949.trk	203418.trk
102311.trk	130114.trk	150423.trk	171633.trk	187345.trk	204521.trk
102816.trk	130518.trk	155938.trk	172130.trk	191033.trk	205220.trk
104416.trk	131217.trk	156334.trk	173334.trk	191336.trk	209228.trk
105923.trk	131722.trk	157336.trk	175237.trk	191841.trk	212419.trk
108323.trk	132118.trk	158035.trk	176542.trk	192439.trk	214019.trk
109123.trk	134627.trk	158136.trk	177140.trk	192641.trk	214524.trk
111312.trk	134829.trk	159239.trk	177645.trk	193845.trk	221319.trk
111514.trk	135124.trk	162935.trk	177746.trk	195041.trk	233326.trk
114823.trk	137128.trk	164131.trk	178142.trk	196144.trk	239136.trk
115017.trk	140117.trk	164636.trk	178243.trk	197348.trk	246133.trk
115825.trk	144226.trk	165436.trk	178647.trk	198653.trk	249947.trk
116726.trk	145834.trk	167036.trk	180533.trk	199655.trk	251833.trk
118225.trk	146129.trk	167440.trk	181232.trk	200210.trk	257845.trk
125525.trk	146432.trk	169040.trk	182436.trk	200311.trk	263436.trk
126426.trk	146735.trk	169343.trk	182739.trk	200614.trk	
126931.trk	146937.trk	169444.trk	185442.trk	201515.trk	

References

Devereux BJ, Clarke A, Marouchos A, Tyler LK (2013) Representational similarity analysis reveals commonalities and differences in the semantic processing of words and objects. J Neurosci 33(48):18906-18916. https://doi.org/10.1523/JNEUROSCI.3809-13.2013

Devinsky O, Farah MJ, Barr WB (2008) Visual agnosia. Handbook of clinical neurology, 88, pp 417-427. https://doi.org/10.1016/S0072-9752(07)88021-3

Ferreira CT, Ceccaldi M, Giusiano B, Poncet M (1998) Separate visual pathways for perception of actions and objects: evidence from a case of apperceptive agnosia. J Neurol Neurosurg Psychiatry 65(3):382-385. https://doi.org/10.1136/jnnp.65.3.382

Gutschalk A, Uppenkamp S, Riedel B, Bartsch A, Brandt T, Vogt-Schaden M (2015) Pure word deafness with auditory object agnosia after bilateral lesion of the superior temporal sulcus. Cortex 73:24-35. https://doi.org/10.1016/j.cortex.2015.08.001

Humphreys GW, Riddoch MJ (2006) Features, objects, action: The cognitive neuropsychology of visual object processing, 1984–2004. Cogn neuropsych 23(1):156-183. https://doi.org/10.1080/02643290542000030

Matchin W, den Ouden DB, Hickok G, Hillis AE, Bonilha L, Fridriksson J (2022) The Wernicke conundrum revisited: evidence from connectome-based lesion-symptom mapping. Brain 145(11),:3916-3930. https://doi.org/10.1093/brain/awac219

Niessen E, Fink GR, Weiss PH (2014) Apraxia, pantomime and the parietal cortex. Neuroimage: cli 5:42-52. https://doi.org/10.1016/j.nicl.2014.05.017

Patterson K, Nestor PJ, Rogers TT (2007) Where do you know what you know? The representation of semantic knowledge in the human brain. Nat Rev Neurosci 8:976–987. https://doi.org/10.1038/nrn2277

Taylor JSH, Rastle K, Davis MH (2013) Can cognitive models explain brain activation during word and pseudoword reading? A meta-analysis of 36 neuroimaging studies. Psychol Bull 139(4):766. https://doi.org/10.1037/a0030266

Vuilleumier P, Henson RN, Driver J, Dolan RJ (2002) Multiple levels of visual object constancy revealed by event-related fMRI of repetition priming. Nat Neurosci 5(5):491-499. https://doi.org/10.1038/nn839
